# The Case for Local AI Development: Lessons From Computer‑Aided Detection of Tuberculosis and Silicosis in Southern Africa’s Ex‑Miners

**DOI:** 10.5334/aogh.5064

**Published:** 2026-02-12

**Authors:** Sean Terespolsky, Annalee Yassi, Rodney Ehrlich, Joshua Bruton, Karen Lockhart, Hairong Wang, Richard Klein, Warrick Sive, John Statheros, Jerry M. Spiegel

**Affiliations:** 1 School of Computer Science and Applied Mathematics, University of the Witwatersrand, Johannesburg, South Africa; 2 School of Population and Public Health, University of British Columbia, Vancouver, BC, Canada; 3 Division of Occupational Medicine, School of Public Health, University of Cape Town, Cape Town, South Africa; 4 School of Clinical Medicine, Faculty of Health Sciences, University of the Witwatersrand, Johannesburg, South Africa

**Keywords:** digital colonialism, artificial intelligence, computer‑aided detection (CAD), tuberculosis (TB), silicosis, local development

## Abstract

The co‑epidemic of silicosis and tuberculosis (TB) in South Africa’s mining industry affects a large number of migrant workers and is compounded by limited access to chest X‑ray (CXR) screening. Although artificial intelligence (AI)‑based computer‑aided detection (CAD) systems for TB have demonstrated impressive accuracy against microbiological standards, validation among silica‑exposed populations has been limited. Moreover, well‑documented biases hinder CAD utility in diverse patient populations, potentially exacerbating existing healthcare inequities. In this article, we describe the challenges in developing CAD systems for TB and silicosis and present the potential benefits local public‑sector development initiatives can bring.

Using a local dataset of 2000 CXRs from silica‑exposed Southern African mineworkers, alongside publicly available international datasets and pretrained CAD models, we present empirical evidence of CAD biases. Dimensionality reduction analysis produced visual mappings that demonstrate how local CXRs form a distinct cluster, separate from international images. We also found that, relative to TB, reducing image resolution disproportionately degraded silicosis detection. Further visualizations proved that accuracy metrics alone are insufficient measures of clinical reliability, possibly obscuring deployment failures.

We conclude that local public‑sector CAD development offers a viable alternative to reliance on externally developed systems that likely exclude underserved populations. Addressing CAD deficiencies requires curating population‑representative datasets that capture local epidemiology and transparent, open‑source development practices that enable peer review and bias correction. Embedding technical and clinical expertise locally can transform AI‑based CAD from a potential instrument of digital colonialism into a mechanism that produces contextually appropriate diagnostics while advancing knowledge for equitable AI deployment worldwide.

Despite enthusiasm about the potential of artificial intelligence (AI) to advance global health equity [1, 2], meeting the needs of underserved groups remains challenging [1, 3]. Indeed, it has been suggested that global power relations in AI development risk exerting pressures that run contrary to equity [4]. In this article, drawing on evidence from over seven years of research on computer‑aided detection (CAD) for silicosis and tuberculosis (TB) in ex‑gold miners in Southern Africa [5–9], we identify several challenges and suggest ways to optimize use of this technology. Specifically, we analyse technological and structural challenges of relying exclusively on international or commercial AI and consider how local, public‑sector development of AI‑based CAD solutions could address these impediments for mining and ex‑mining populations.

## 1. Background

Given the belated recognition that about half of TB episodes are asymptomatic, the World Health Organization (WHO) has recommended the use of chest X‑rays (CXRs) for TB screening and triage [10]. At the same time, commercially available CAD systems for CXR readings have demonstrated high diagnostic performance [2, 10, 11]. Accordingly, WHO endorses the application of CAD for TB screening and triage in place of human readers, albeit with a low certainty of evidence [10]. This development raises the question of the conditions that would allow CAD to attain its full global health potential [12]. In the setting of interest here, current CAD systems have achieved diagnostic discrimination scores (as measured by the area under the curve [AUC] of the receiver operating curve) above 90% (>98% in the best performing system) when tasked with identifying any abnormality; TB only (CXR only, without microbiological confirmation); silicosis only or combined disease only against normal images [9]. However, when tasked with detecting TB only (as defined above) in a case‑mix that included TB, silicosis and combined disease, the AUC dropped to 81% in the best performing system. This finding highlights the challenge to CAD when faced with overlapping radiological features in multi‑morbidity populations [9]. More generally, in a non‑mining population of South Africans in which silicosis was not a significant feature and TB was defined microbiologically, the AUC performance of a number of CAD systems deteriorated significantly when applied to individuals >55 years, as well as those with previous TB [11].

Race‑based employment practices began in South Africa’s (SA’s) early gold mining industry [13, 14] and relied on migrant Black workers from rural SA and neighbouring colonies (later states). By the end of the 20th century, many of these miners were afflicted by a triple epidemic of silicosis, TB and HIV, despite some reduction in mine dust levels [13, 15]. Given that silica dust exposure, even without silicosis—the fibrotic lung disease caused by silica inhalation [16]—increases the lifelong risk of TB [17], working and living conditions during that era intensified TB transmission [17]. As a result, TB rates among gold miners were two to three times greater than those of the general population [18]. When migrant workers returned home, they, as well as their households and communities, remained at risk from undetected or inadequately treated TB, including drug‑resistant TB [19].

Populations exposed to silica have therefore been identified by the WHO for systematic TB screening [10]. However, silicosis produces lung changes that may mask or mimic the appearance of TB on CXR, and vice versa, complicating detection [20]. For active and former miners, distinguishing silicosis from TB has crucial implications for both healthcare management and compensation eligibility [14]. Consequently, in respect of both diseases, assessing CAD performance in local, silica‑exposed populations is needed to ensure reliable screening of mineworkers.

To date, existing TB CAD systems have been trained on populations where the co‑occurrence of silicosis is low or absent. Nonetheless, the limits to generalizability of such systems have been demonstrated even outside relevant multi‑morbidity settings. In a recent evaluation of 12 internationally developed TB CAD systems applied to a sample of the general South African population, in which silica exposure can be assumed to be low, models exhibited poor specificity (negative case identification rate) when operating at the WHO target sensitivity (positive case identification rate) levels for screening [11]. Incorporating additional covariate information (such as age, sex and work history) showed mixed effects across the evaluated models, with marginal performance gains for some systems but not others.

The above considerations carry additional social implications. Despite SA’s democratic transition in 1994, disparities in access to medical evaluation for occupational lung disease, correlated with race and country of origin [14, 21, 22], have persisted. The inability to effectively address these disparities has left hundreds of thousands of former miners with limited social protection and access to screening [14, 23], with barriers highest for migrant workers [7]. Even beyond the current burden of disease, the need for TB, silicosis and other occupational lung disease detection is likely to continue as the mining industry expands throughout Africa.

Given the shortage of trained health professionals to detect occupational lung disease in rural and remote areas, efforts were initiated in SA to explore the feasibility of applying CAD solutions. However, only one commercially developed CAD system in the peer‑reviewed literature has shown reasonable accuracy in detecting silicosis in this multi‑morbidity setting to date [6]. Accordingly, our international collaboration turned to focus on assessing the feasibility and potential benefits of publicly supported local development of CAD, including what could be learned through the development cycle, particularly in systems training.

## 2. The Limitations of International CAD Systems

### 2.1 Generalization failures: Domain overfitting & shortcut learning

The promise of AI‑based CAD systems in general to support accurate and scalable diagnostics in low‑resource healthcare settings is limited by well‑documented biases in medical imaging AI [24]. When the population used for model training differs from the target population—whether due to demographics, disease manifestations or imaging protocols—diagnostic performance predictably deteriorates, often with uneven impacts across patient subgroups [25]. This phenomenon, described as ‘domain‑overfitting’ [26], occurs when systems memorize training‑specific patterns rather than learning generalizable disease features. Consequently, models that demonstrate strong performance on validation data from similar populations fail to maintain comparable levels of accuracy or fairness when applied to heterogeneous, out‑of‑distribution populations, with the greatest harm typically borne by marginalized groups [27].

Moreover, imaging models frequently infer and internalize demographic and non‑clinical information from CXRs, including age, race and sex, subsequently using these and other spurious correlations for prediction rather than focusing on true pathology—a process known as ‘shortcut learning*’* [28]. This pattern extends across imaging modalities, where models exploit easily detectable but clinically irrelevant features (such as imaging device markers) instead of disease‑specific patterns [29]. These shortcuts can lead to systematic biases against historically underserved subgroups, producing unreliable predictions when models encounter new demographic contexts or co‑occurring conditions [27]. The described mechanisms highlight that reported accuracy alone may mask failures in diagnostic reliability, especially when deployed in low‑ and middle‑income countries (LMICs) where imaging protocols (including scanner equipment and operating technicians), disease patterns and clinical workflows are likely to differ markedly from those in high‑income countries (HICs).

### 2.2 Representation gaps: Dataset shifts & imbalance

Systematic inequities in global dataset development exacerbate cross‑population generalization failures. Over half of publicly available medical imaging datasets originate from just two countries, namely the US and China, with populations from the majority of LMICs under‑represented in dataset development and research authorship [30]. Accordingly, disparities in disease prevalence between LMICs and HICs are likely to result in systematic under‑representation of important diagnostic groups in accessible datasets.

As noted above, in the Southern African occupational health context, the scarcity of silicosis representation in global CXR datasets is a barrier to the development of effective CAD for silicosis and related TB. Most global repositories are assembled through ease of access to images, without explicit regard for potential confounders that local experts recognize. This limitation manifests as both a ‘domain shift’—resulting from variations in imaging protocol and population demographics—and a more fundamental ‘label shift’*,* where silicosis as a diagnostic group is largely absent in training data. Subsequently, when algorithms are trained on datasets with such sparse representation of groups (known as ‘imbalanced data’), the systems tend to approximate the statistical trends of conditions they are more likely to encounter during training [31]. For silicosis detection in SA miners, this means that CAD will tend to favour predicting more common diagnoses like TB, even when silicosis‑specific features such as diffuse nodulation are present.

### 2.3 Why transfer learning alone is insufficient

A common framework used to address domain and label shifts, as well as data imbalance, is ‘transfer learning*’* (TL). TL makes use of models pretrained on large public datasets and adapts or fine‑tunes them using smaller, local datasets [32]. This approach provides a theoretical way to transfer knowledge across domains and mitigate inherent system biases. However, in practice, TL faces limitations. The scarcity of high‑quality local data, as well as differences in comorbidity prevalence between domains, significantly reduces the ability of TL to achieve satisfactory performance. Without adequate exposure to relevant comorbidities, international models may struggle to distinguish between diseases, causing misclassification.

Prior evidence indicates that biases encoded during original model training are often retained, or even amplified, during TL [33]. Ultimately, these observations should motivate us to move beyond adaptation‑based paradigms and towards the full development of CAD systems grounded in local context and representative information.

### 2.4 Empirical evidence from the southern african mining context

In collaboration with the state compensation agency, the South African Medical Bureau for Occupational Diseases (MBOD), we conducted preliminary experiments that provide empirical evidence of the biases and limitations discussed above. Using approximately 2000 anonymized CXRs from silica‑exposed miners across Southern Africa—whose claims had recently been assessed by the MBOD Certification Committee—and two open‑source international CXR datasets [34, 35], we examined CAD behaviour in the local context. In the MBOD dataset, TB and silicosis labels reflect radiological adjudication within the established MBOD clinical workflow. Microbiological confirmation is reserved for suspected active TB and is not clinically informative for the predominantly inactive TB and silicosis cases represented in this population. When considered alongside the international datasets, our investigations produced three key insights.

**Figure 1:**
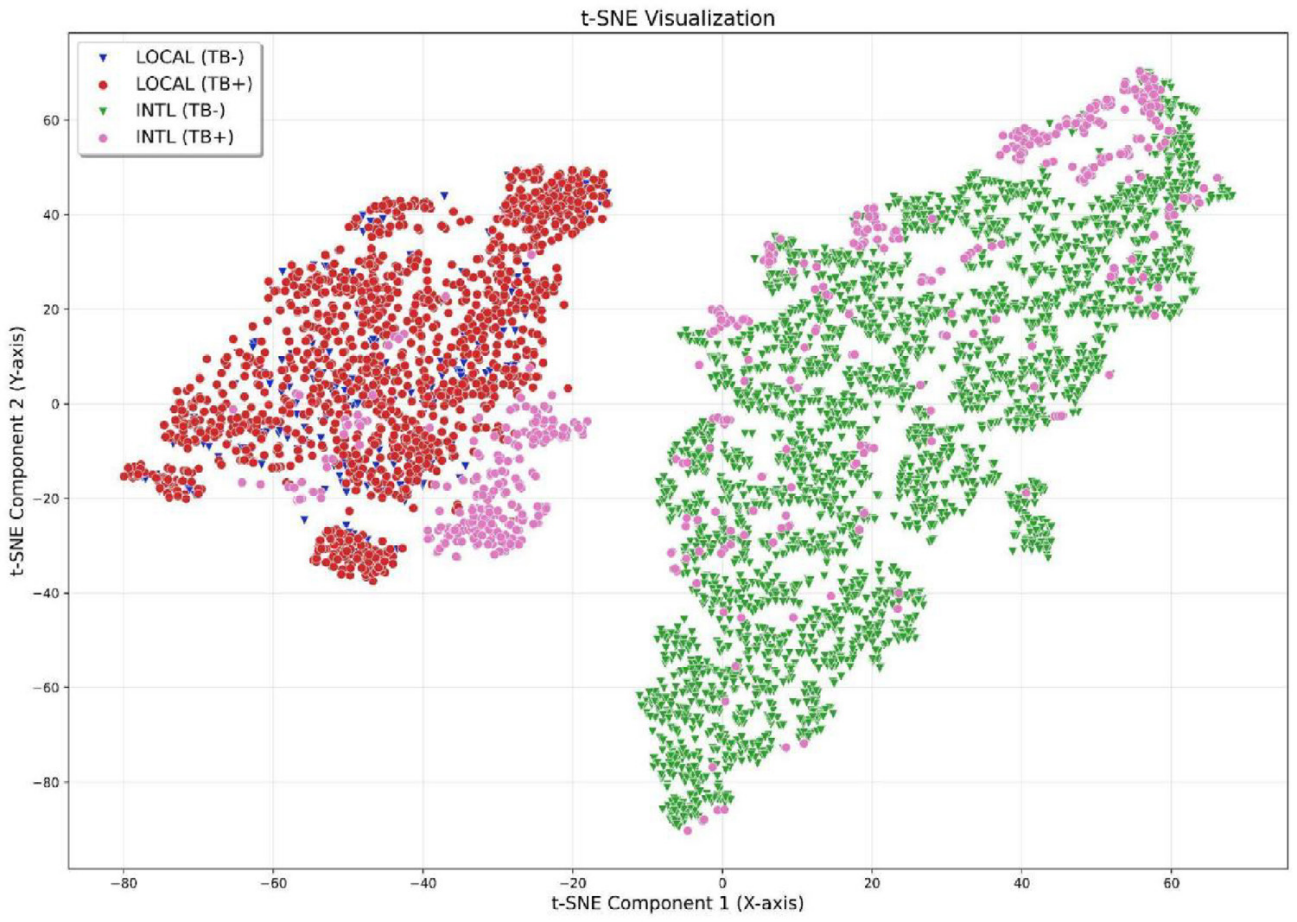
Visual comparison of CXR datasets. t‑SNE visualization showing how a public, internationally trained CAD model [36] encodes CXRs from local and international datasets. Each point represents a single CXR: blue triangles and red dots indicate local TB‑negative and TB‑positive samples, respectively; green triangles and pink dots indicate international TB‑negative and TB‑positive samples [34]. The local dataset is labelled ‘LOCAL’ and the international dataset is denoted ‘INTL’ (top‑left legend). The axes are considered abstract coordinates and can be simply thought of as *X* and *Y* coordinates; distances between markers reflect the model’s perceived similarity between CXRs. In the international dataset, positive and negative cases appear well separated, showing the model’s ability to discriminate TB status in populations similar to those seen during training. In contrast, local CXRs form a single cluster, with poor separation between positive and negative cases. Essentially, local images are viewed as a single group that differs substantially from positive and negative international TB cases alike. A small subset, all TB positive, of the international sample lies near the local images. Local TB‑negative samples would therefore be interpreted as more similar to international TB‑positive CXRs than to international TB‑negative cases. The inference is that the model’s TB‑negative baseline, learned from international data, does not generalize appropriately to the local population. In a truly generalizable CAD model, diagnostic groups would separate across populations and datasets: negative cases from all sources would cluster together, while the positive cases form a distinct group. This would indicate that a model has likely learned clinically relevant features instead of dataset‑specific artefacts.

First, we utilized an international, publicly available CAD model [36] to demonstrate how biases can manifest in practice. By applying a dimensionality reduction method called t‑Distributed Stochastic Neighbour Embedding (t‑SNE), we created a visual ‘map’ that shows how the model groups and distinguishes between different images for TB. In Figure 1, each point represents one CXR, and the relative distances between points reflect how similar or dissimilar the model perceives the images to be. Colours indicate dataset origin, while shapes denote disease status: blue and red markers represent CXRs from local miners, whereas green and pink markers correspond to CXRs from an open‑source international TB dataset [34]. Notably, the international TB dataset encompasses portions of open‑access imaging sets from various regions (including North America, Europe and Asia) but does not contain any CXRs from African populations. The local dataset, in turn, is characterized by a preponderance of TB‑positive images, consistent with its origin in a compensation‑agency cohort of miners afflicted with a large TB disease burden.

Ideally, a robust CAD system should consistently separate positive and negative cases irrespective of population. However, Figure 1 shows that the local CXRs form a distinct cluster, largely separate from the international images—indicating that the model perceives the local images as different even when both datasets contain TB‑positive cases. The exception is a small subset of TB‑positive CXRs (pink dots) from the international dataset that overlap or lie near the local cluster. Implicitly, the model perceives this subset as similar to both TB‑negative and TB‑positive local CXRs alike, a problem likely due to a lack of exposure to local epidemiology.

Within the international cluster, images appear well separated by TB status, indicating that the model has captured disease‑related differences in populations similar to its training data—a pattern that can be considered qualitatively consistent with good TB detection performance. By contrast, this separation is absent among the local images, where positive and negative samples lie in close proximity. This suggests that the features previously learned by the model do not generalize effectively across diverse domains, where variations arising from domain shift can exceed those associated with the target disease classes. Consequently, the system’s practical utility may be limited in populations that are atypical relative to the training cohorts.

These observations highlight how high regional prevalence of comorbidities—such as silicosis and post‑TB lung disease in our case—can contribute to visual and statistical variations in TB CXRs across populations. Consequently, without sufficient exposure to local TB samples, the model struggles to equally discriminate TB manifestations across regions, instead focusing on region‑specific differences between the datasets.

For clarity, the figure has included only TB labels due to the lack of available international CXR datasets with silicosis presence. We have relied on qualitatively contrasting what is observed by applying the quantitatively grounded t‑SNE statistical method. Application of conventional inferential statistics is not feasible in this context due to small dataset size, incompatibility of models with different disease characteristic distributions and the complexities of deep learning features.

Second, we discovered that the fine‑grained radiological features of silicosis compounded model limitations. Our findings (which will be detailed in subsequent work) revealed that a reduction in image resolution disproportionately degraded silicosis detection performance, while TB detection was significantly less affected. Overall, halving the image resolution led to as much as a 5% drop in silicosis detection accuracy, while TB detection performance either remained unchanged or marginally improved. This suggests that international CAD systems, optimized for broad populations and visually prominent diseases, may perform well on common targets but fail to capture subtle, locally relevant disease markers. When coupled with domain and label shifts, these impediments can lead to systematic misclassification of both positive and negative cases, with direct implications for health equity and patient care.

Finally, to assess the reliability of imported pretrained systems, we used attention map visualizations, which are a form of explainable artificial intelligence [37]. This technique indicates the regions of the CXR that contributed strongly to the model’s predictions. For these experiments, only an international dataset [35] was used, as the local CXRs lack the necessary labels for this type of analysis.

**Figure 2:**
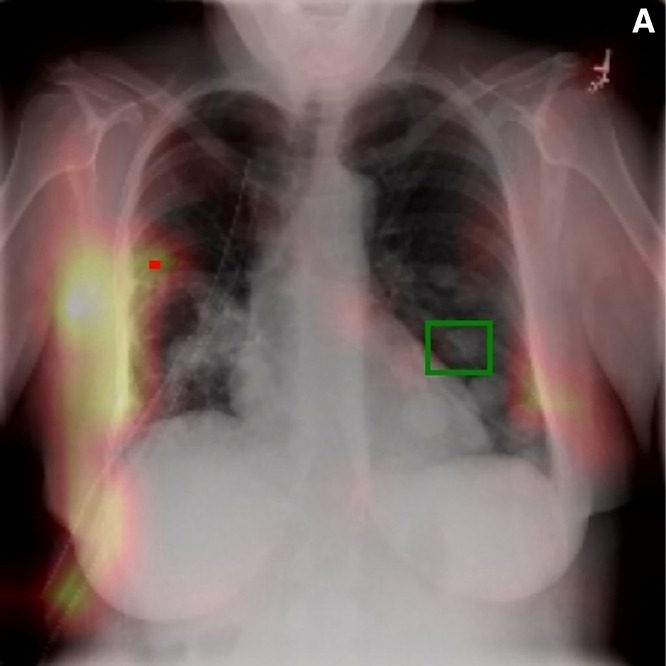

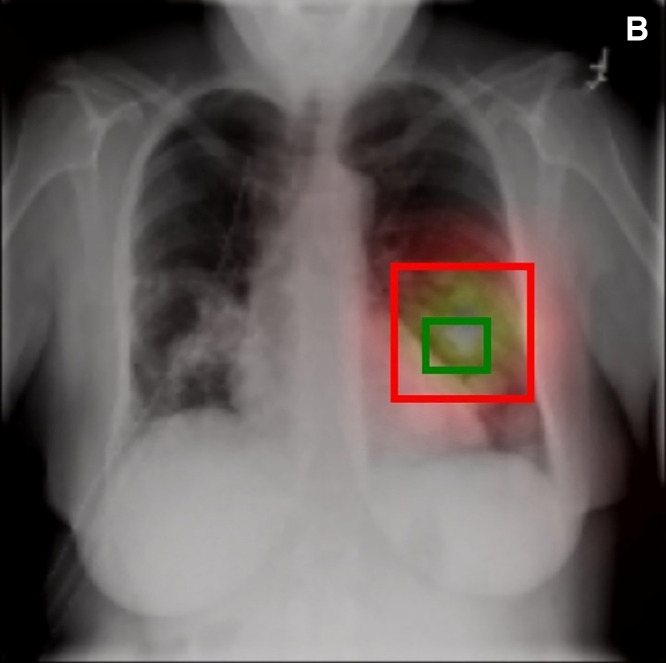

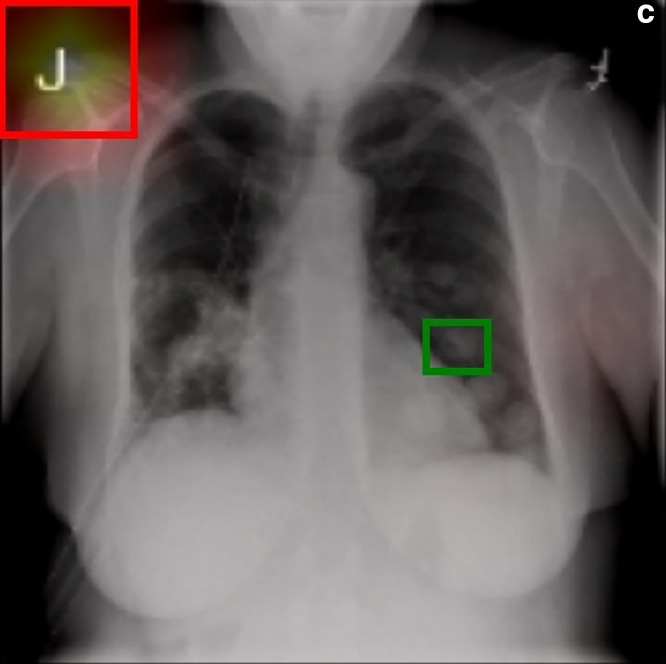
Visual attention maps showing the CXR regions the model paid most attention to when making predictions. Areas with red markers are regions of maximum model attention, while regions marked in green are the true locations of nodules. **(A)** Model A (diagnostic accuracy 80.14%): In this example, maximum model attention is in a different lung from the true location, with very little model focus on the correct image region. **(B)** Model B (diagnostic accuracy 80.22%): In this example, maximum model attention and true location coincide. The entire true location is encompassed by the region the model relied on for its diagnostic prediction. **(C)** Model C (diagnostic accuracy 95.46%): In this example, maximum model attention is on the ‘J’ image marker in the top right corner. This situation exemplifies shortcut learning in practice, where high reported accuracy can obscure diagnostic failures.

In Figure 2A‑C, the attention heatmap produced by each model is superimposed on the diagnosed CXR. The red rectangular markers indicate the model’s points of maximum attention, while green rectangular markers show the true locations of nodules. Each figure corresponds to a different model (A, B and C), presented in order of increasing diagnostic performance (80.14%, 80.22% and 95.46%). While model A and model B achieve nearly identical performance, only the latter focuses on true disease marker location. Model C, despite substantially higher diagnostic performance, relies almost entirely on an image‑specific artefact rather than clinically meaningful features.

Such an undesirable pattern exemplifies shortcut learning in practice, showing that diagnostic performance can mask reliance on spurious correlations. Notably, lower performing models can still maintain clinically sound feature focus. This suggests that accuracy metrics alone are insufficient measures of diagnostic reliability, motivating further investigation into ways of assessing clinical validity alongside model performance.

## 3. Local Development as a Path to Health Sovereignty

The findings outlined in Section 2 suggest potential failures that could perpetuate healthcare disparities [38, 39]. We next examine broader principles that further argue for locally led, public‑sector‑based, transparent approaches to address these and other limitations of relying exclusively on internationally developed commercial products.

### 3.1 Addressing structural pitfalls of imported systems

It is increasingly being demonstrated that systematic failures of imported, internationally developed CAD systems may reproduce a pattern of what has been labelled ‘digital colonialism’, wherein data from LMICs are extracted, commodified and used to advance global innovation, while yielding little benefit for the communities from which they originate [4, 40]. In this process, local expertise remains peripheral, constrained to supplying information rather than shaping priorities or outcomes, reflecting the ‘paradox of participation’ [41].

Furthermore, commercial incentives likely lead to scalability and profit being prioritized over equity and contextual relevance, systematically distorting research priorities and deployment to favour market expansion instead of local health needs [41]. For instance, diseases such as silicosis—highly prevalent in some locales but relatively rare globally—offer limited commercial returns, making them unattractive to private developers. As a result, CAD for silicosis can be regarded as a public good in the economic sense. With only about 1% of global health data originating from African populations—despite considerable diversity and urgent local health needs—imported AI systems are rarely calibrated to local epidemiological realities [42], for reasons presented above. Consequently, these tools risk not only amplifying misdiagnoses but also constraining the development of the scientific and technical sovereignty needed for sustainable, contextually relevant health innovation.

### 3.2 Open science: Building reliability & trust

Given global structural inequities, transparent open science becomes an important goal. A significant proportion of published CAD‑related results are difficult to replicate, creating scepticism among users [43]. Open science practices—including sharing of datasets, models and methodological frameworks—create avenues for iterative refinement and independent validation, supporting continuous improvement in ways that proprietary systems cannot. Transparency is especially important in low‑resource, high‑burden regions, where access to qualified radiologists and healthcare professionals is limited and imported technologies need to be continuously tested and adapted for local effectiveness [43]. Furthermore, the open reporting of model limitations and biases is more likely to cultivate trust among local healthcare workers, policymakers and affected communities [3].

As noted above, models optimized solely for global accuracy often fail when deployed in new or underserved settings, leading to unreliable predictions [27, 29, 42]. These failures may be invisible upfront in proprietary systems, where negative results and context‑specific limitations are rarely disclosed [44]. Open and transparent approaches allow local knowledge‑users to partner with system developers. This collaboration can improve the adaptation of algorithms to regional epidemiology and ensure that diagnostic performance reflects the realities of the populations served [4, 10, 42].

Without the commercial pressures to publicize positive results, local public‑sector teams can transparently report model limitations and failures, validate systems under real‑world conditions and share methodologies for peer review [44]. This alignment with clinical research principles enables identification of algorithmic bias, fostering a self‑correcting ecosystem that produces both technically robust and contextually appropriate public health tools. An example outside health systems is the *Masakhane* initiative’s participatory approach to African language datasets [45] in which locally led, community‑engaged approaches support model sustainability, ownership and continual improvement—principles that are directly applicable to locally led CAD initiatives.

### 3.3 Beyond accuracy, towards reliability

Another concern, unrelated to reporting bias, is that even where CAD systems show high diagnostic performance metrics, these may overstate the true clinical reliability of such tools—particularly in diverse or under‑represented populations. Consistent with existing literature, our visualizations of model attention (seen in Section 2.4) demonstrate how CAD models may exploit dataset‑specific artefacts to achieve ostensibly strong performance rather than genuinely identifying disease markers [29, 46]. Even when validated on external datasets, CAD models may continue relying on shortcuts that can persist across datasets without degrading headline metrics, as has been shown previously for Covid‑19 CXRs [47].

Multi‑society guidelines have emphasized that truly reliable CAD demands detailed reporting of subgroup‑specific performance and failure modes—criteria often absent from the commercial AI landscape [48]. Further research has highlighted how, even in deployed systems, deficiencies in publishing clinical evidence and reporting known limitations persist [49]. In most reported cases, vendors omitted evaluations that may reveal shortcut learning or cross‑population failures—perpetuating the use of systems that appear functional while fundamentally misunderstanding the clinical task.

Such reliability failures have both economic and public health implications. While commercial vendors may claim, for example, that economies of scale make their solutions cost‑effective, the above findings suggest that such savings may be illusory if models misdiagnose the populations they are meant to serve—ultimately inflating downstream costs through inappropriate decisions and missed public health interventions. Together with our attention to the findings and visualizations presented above (Section 2.4), such concerns support the argument for locally driven development pipelines.

## 4. Concluding Remarks: The Path Forward

Our findings show that image resolution, disease localization and contextual factors strongly shape CAD performance. These insights underscore the need for full model development using locally relevant data, rigorous validation and attention to diagnostically meaningful features—prioritizing reliability over raw accuracy alone. Commercial vendors can acquire local data but may face a misalignment between profit motives and public health needs, limiting region‑specific adaptations and risking the perpetuation of digital and clinical inequities. Even when local data are purchasable, diseases such as silicosis offer little commercial incentive for proprietary development. Silicosis CAD is thus essential for local health equity. However, development in this space is unlikely to emerge from many profit‑driven models.

A sustainable path forward lies in locally led and publicly accountable initiatives grounded in equity, transparency and open science. By investing in local expertise, developing open‑access representative datasets, embedding continuous validation and openly reporting limitations, health systems can assert agency over AI diagnostics. Cross‑sector local collaboration—with partners familiar with the relevant clinical epidemiology—can ensure that the necessary context becomes a design principle rather than an afterthought.

The Southern African mining population experience offers valuable lessons for other LMICs and high‑burden regions alike, strengthening the potential of AI and CAD to serve as sustainable, contextually relevant public health assets rather than merely imported commodities—simultaneously reducing dependence while supporting the availability of appropriate diagnostics that meet local needs.
